# Exercise addiction in Spanish athletes: Investigation of the roles of gender, social context and level of involvement

**DOI:** 10.1556/JBA.2.2013.4.9

**Published:** 2013-12-13

**Authors:** Attila Szabo, Ricardo De La Vega, Roberto Ruiz-BarquÍn, Oswaldo Rivera

**Affiliations:** ^1^Eötvös Loránd University, Budapest, Hungary; ^2^Autonomous University of Madrid, Madrid, Spain

**Keywords:** exercise dependence, exercise volume, individual, group, prevalence, sport

## Abstract

*Background and aims:* In nomothetic research exercise addiction is studied on the basis of symptoms which are most often linked to exercise volume. However, other factors may also affect individuals' susceptibility to the disorder. The aim of this research was to examine the influence of gender, social context (team or individual sport), and level of athletic training on symptoms of exercise addiction. *Methods:* Two groups of university athletes – sport- (*n* = 57) and non-sport orientation (*n* = 90) – and a group of elite ultra-marathon runners (*n* = 95) completed the Exercise Addiction Inventory (EAI). The psychometric properties of the Spanish EAI were determined. *Results:* EAI scores were higher in men than women (*p* = .018). Participants in team sports reported higher EAI scores than individual athletes (*p* = .005). Elite runners scored higher on the EAI than university athletes (*p* = .005), but their scores were unrelated to the volume of training. The prevalence of “at risk” for exercise addiction was 7%–10% in university athletes and 17% among the ultra-marathon runners. The Spanish EAI showed good psychometric properties. *Discussion:* The results of the current inquiry show that several factors – including gender, level of athletic training, and social context of the training – affect exercise addiction and, in line with the literature, the volume of exercise did not emerge as an index of susceptibility to exercise addiction.

## INTRODUCTION

Exercise addiction (EA) may be conceptualized as the loss of control over one's exercise behavior that becomes an obligation in which the classical symptoms of addictions are also manifested (Berczik et al., 2012; Szabo, 2010). In lack of exercise, the affected individual experiences severe psychological discomfort. Disordered exercisers are distinguished from other high-volume exercisers, like elite athletes, because the latter do not encounter negative consequences as a results of their exercise (Berczik et al., 2012).

The prevalence of EA is relatively rare. Using the Exercise Addiction Inventory (EAI; Terry, Szabo & Griffiths, 2004), a recent inquiry has revealed that about 3.2% of the habitual exercisers and 0.5% of the general population may be at risk of EA (Mónok et al., 2012). The figure, however, may vary in accord with several – yet unclear – factors. Szabo and Griffiths (2007) showed that the risk for EA was near 3.6% in habitual exercisers, while the figure was almost double (6.9%) in sport science university students. The risk in runners was found to be 22% (Anderson, Basson & Geils, 1997) and competitive runners were more at risk (50%) than non-competitive runners (Smith, Wright & Winrow, 2010). However, a study of French ultra-marathoners (Allegre, Therme & Griffiths, 2007) revealed that only 3.2% of the runners were at risk for EA.

Systematic investigation of risk for EA in athletes in team and individual sports was not reported to date. However, a recent study found no difference between fitness and soccer athletes (Lichtenstein, Christiansen, Bilenberg & Støving, 2012). Since fitness exercises are often performed in group, better understanding of EA in individual and team sports is needed. Actual cases of EA may be greater in the former due to the liberty of need-based scheduling.

Furthermore, some inquiries have disclosed gender differences in EA (Hausenblas & Downs, 2002; Pierce, Rohaly & Fritchley, 1997; Tata, Fox & Cooper, 2001) while others did not (Furst & Germone, 1993; Modolo et al., 2011). These finding are also controversial because differences were in opposite directions (Hausenblas & Downs, 2002 Lejoyeux; Pierce et al., 1997). Therefore, further examination of gender differences in EA is warranted.

The present study investigated moderators contributing to variability in susceptibility to EA. Specifically, based on past inquiries (Allegre et al., 2007; Szabo & Griffiths, 2007) the proneness to EA was examined in Spanish sport science and non-sport orientation university athletes and a group of elite ultra-marathon runners. Considering the controversial reports in the literature, gender differences in EA were further investigated. Finally, the risk of EA in a social context, in terms of individual or group exercises, was also explored.

## METHODS

### Participants

Non-sport oriented (*n* = 90) and sport science (*n* = 57) university athletes, involved in team and individual sports, were invited to participate in the study. Elite ultra-marathoners (*n* = 95) were also solicited to participate in the inquiry. A total of 242 athletes (164 men and 78 women) were recruited from Madrid Metropolitan area (Spain) and Autonomous University of Madrid (*M*_age_ = 27.54 yrs; *SD* = 10.65). They reported training an average of 6.71 h (*SD* = 3.53) per week.

### Materials

The Spanish version of the 6-item Exercise Addiction Inventory (EAI; Terry et al., 2004) was used along with a demographic questionnaire. The EAI comes with good psychometric properties (Monók et al., 2011; Terry et al., 2004). The properties of the Spanish scale are reported in the Results section.

### Procedure

Participants completed the questionnaire in a natural setting (university or training venues) in the presence of an experimenter. Data collection lasted two months. Data were entered in Excel files then imported into the SPSS software for statistical analyses.

### Ethics

Participants were given informed consent about the study that was approved by the local Ethics Board at the Universidad Autónoma de Madrid. The research was conducted in full agreement with the ethical principles for research with human subjects of the Helsinki Declaration (World Medical Association, 2008) and the guidelines for ethical considerations in psychology research with human participants (British Psychological Society, 2010).

## RESULTS

The Spanish version of the EAI was subjected to factor analysis. Each of the six item was statistically significantly correlated (*p* < .001) with another item, supporting factorability. The Kaiser-Meyer-Olkin (KMO) measure of sampling adequacy was .801. The Bartlett's test of sphericity was significant (χ^*2*^ (15) = 253.08, *p* < .001). Diagonals of anti-image correlation matrix were all over .77 supporting the inclusion of each item in the factor analysis. Principle component analysis was used and only components with eigenvalues of =1.0 were retained. In this way one factor emerged that accounted for 41.99% of the total variance. A minimum loading of 0.40 was observed for each item. The internal reliability of the Spanish scale was (Cronbach's alpha) .71. Construct validity was determined by comparing median-split groups of high- (above 6 h/week) and low-exercise volumes (below 6 h/week) groups. The two groups differed statistically significantly in EAI scores (*F* (1, 201) = 23.14, *p* < .001).

Group differences in EAI scores were tested with group (3) by gender (2) analysis of variance (ANOVA). The test yielded two main effects; one for gender (Means: men = 19.40, *SD* = 3.50, and women 18.23, *SD* = 3.73; *F* (1, 236) = 5.08, *p* = .03, effect size (Cohen's *d*) = .32), and one for group (*F* (2, 236) = 6.81, *p* = .001). For the latter, Tukey's HSD post hoc tests showed that elite runners (EAI: *M* = 20.08; *SD* = 3.70) differed from university athletes (*M* = 18.41, *SD* = 3.46, and *M* = 18.23, *SD* = 3.34, respectively, *p* = .005, the effects sizes *(d)* were .47 and .52, respectively). The two groups of university athletes did not differ from each other and the group by gender interaction was statistically not significant.

Differences in EAI between athletes in individual and team sports included data from university students only who did not differ from each other on the previous test. The ANOVA showed that athletes in team sports scored higher on EAI than individual athletes (*M* = 19.12, *SD* = 3.34, and *M* = 17.55, *SD* = 3.15; *F* (1, 1145) = 8.26, *p* = .005, *d* = .47).

**Figure 1.**
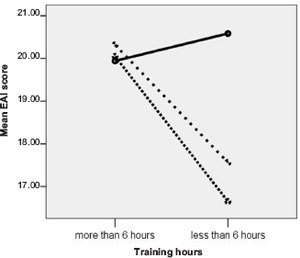
EAI scores of those who train more and less than 6 hours per week

*Note:* Dotted lines represent university athletes and continuous lines represents the elite runners.

To test the link between amount of exercise and EAI scores, a median split was used to generate high- (>6 h/week) and low-exercise-volume (<6 h/week) groups. Excluding those right on the median, the ANOVA revealed a group by volume interaction (*F* (2, 197) = 4.45, *p* = .013). The interaction showed that lesser training is linked to lower EAI scores in university athletes but not in elite runners (Figure 1). The correlation between weekly hours of training and EAI scores was *r* = .24, *p* <.001, *r^2^* = .57.

Finally the prevalence of “at risk” for EA was calculated on the bases of the EAI cut off score of 24 (Terry et al., 2004). In the current research 7% of the sport science athletes, 10% of non-sport science university athletes (8.8% of all university athletes), and 17% of the elite runners have scored 24 or above the EAI. From the whole sample 29 (or 12%) athletes scored 24 or more on EAI. The ratio of men was higher than that of women (21 vs. 8), which was statistically significant (χ^*2*^ (4) = 10.79, *p* = .029).

## DISCUSSION

Preliminary results show that the Spanish EAI has good psychometric properties. All six items emerged as a one-factor solution. The internal consistency of the scale was lower than that of the original (Terry et al., 2004) but it was higher than in a Danish study (α = .66; Lichtenstein et al., 2012) and it was slightly lower but comparable to a large population-wide Hungarian study (α = .72; Mónok et al., 2012). The construct validity of the scale was good as based on the method of Terry et al. (2004). The concurrent validity and test–retest reliability of the Spanish EAI remains to be determined.

The current study shows that proneness to EA varies among athletes. Therefore, it seems impossible the establish a universal prevalence value unless specific populations are examined. High variability noted in past studies, examining EA, was seen as methodological shortcoming (Szabo, 2010). However, the current findings may suggest that specific samples may possess some unique characteristics that contribute to the variability in the established preponderance of EA. It is important to stress that risk for EA is *not a diagnosis* and devotion to athletics may inflate the subjective ratings of the EAI items through infiltration of concepts linked to commitment. In reality few professional athletes were diagnosed with EA.

In the current research no differences were disclosed between sport- and non-sport oriented university athletes. The preponderance of risk for EA in these groups was similar to that reported by Szabo and Griffiths (2007), but higher than the figure disclosed for habitual exercisers. It is possible that university athletics bear some features that raise the ratings of the EAI items. However, it is also possible that cultural and/or local habits or sport practices contribute to the observed differences. For example using the French EAI, Lejoyeux, Guillot, Chalvin, Petit and Lequen (2012) found that the prevalence of exercise addiction was nearly 30% among the customers of a Parisian sport shop. These discrepancies need thorough and systematic investigations in the future.

In agreement with past results (Anderson et al., 1997; Smith et al., 2010), the findings are in discord with Allegre et al.'s (2007) results of 3.2% risk of EA in ultra-marathoners. In the two studies the participants were comparable in number and training habits, but different tools of assessments were used. Indeed Allegre et al. classified 61.1% of their sample as *non-dependent symptomatic.* In general, the reported risk for EA among various groups of runners fluctuates to large extent, which shows that other factors than the exercise – or even level of involvement – may be responsible. This issue calls for research attention in future work.

Another important contribution of the current work is that it provides clear results for unlinking exercise volume from EAI scores. Indeed, as visible in Figure 1, while university athletes training more than six hours per week reported higher EAI scores in contrast to those who trained less than six hours, this was not the case with the elite runners. The latter group showed consistently high EAI scores regardless of training volume. However, only a few (*n* = 12) ultra-marathoners trained less than 6 h per week. In spite of this fact, the statistically significant but low correlation showing only 5.6% common variance between training volume and EAI scores justifies the weak link between the latter variables. Szabo (2010) argued that exercise volume alone is not an index of one's susceptibility to EA.

A finding obtained from an exploratory perspective is that team athletes score higher than individual athletes on the EAI. Since addiction, as a psychological morbidity, cannot be lived out in an organized manner (training), it is possible that athletes in team sports interpret some items of the EAI in a confounding way. Indeed, the current results call for a systematic re-investigation of the validity of the EAI in team sports.

In the current study, women scored lower than men on the EAI. These results agree with those observed in some previous inquiries (Cook, Hausenblas & Rossi, 2013; Hausenblas & Downs, 2002; Tata et al., 2001) but are in discord with others (Modolo et al., 2011; Pierce et al., 1997). It has been argued that women score higher on EA scales when there is an accompanied eating disorder (Szabo et al., 2010; Tata et al., 2001). A clearer understanding of the gender differences in risk for EA should take into consideration the presence or absence of eating disorders.

## CONCLUSION

The present study, apart from preliminary evidence for acceptable psychometric properties of the Spanish EAI, has the following contribution: 1) High variability in proneness to EA may be expected on the basis of exercise and sport practices; 2) Athletes in team sports score higher on EA risk assessment than athletes in individual sports, which could be an artifact; 3) Gender differences in EAI rating occur, but since women scored lower than men, in the current work this finding may not be linked to some correlates of EA, like eating disorders, and 4) The volume of weekly exercise is not related to EAI scores in elite runners, demonstrating that EA is not a function of exercise or training intensity.
